# Severe relapse after cessation of immunosuppressive therapy in a patient with co‐occurrence of neuromyelitis optica spectrum disorder and Sjögren's syndrome: A case report

**DOI:** 10.1002/ibra.12175

**Published:** 2024-09-13

**Authors:** Man‐Min Zhu, Zu‐Cai Xu, Chang‐Yin Yu, Hao Huang

**Affiliations:** ^1^ Department of Neurology Affiliated Hospital of Zunyi Medical University Zunyi China

**Keywords:** immunosuppressive therapy, neuromyelitis optica spectrum disorder, relapse child, Sjogren's syndrome

## Abstract

Neuromyelitis optica spectrum disorder (NMOSD) is a group of autoimmune disorders characterized by inflammatory involvement of the optic nerve, spinal cord, and central nervous system. NMOSD is often associated with other autoimmune disorders, including Sjogren's syndrome (SS). While NMOSD typically occurs at a peak in young or older individuals, the coexistence of NMOSD and SS in a youngster is rare. Here, we presented a case of a 14‐year‐old girl with NMOSD and SS who responded well to immunosuppressive therapy but experienced a severe relapse after discontinuation of therapy. We described the clinical course of a case over 8 years, underscoring the importance of long‐term treatment for NMOSD and SS. This case, along with the review of relevant literature, will raise awareness of this type of disease and facilitate early diagnosis and treatment to avoid serious sequelae.

## Graphical abstract

We report a girl with a diagnosis of “neuromyelitis optica spectrum disorder comorbid Sjogren's syndrome” whose condition showed significant improvement after treatment with corticosteroids and immunosuppressive drugs (prednisolone, hydroxychloroquine, and mycophenolate mofetil). However, she developed a relapse and developed a more severe disease due to voluntary discontinuation of the immunosuppressive drugs.

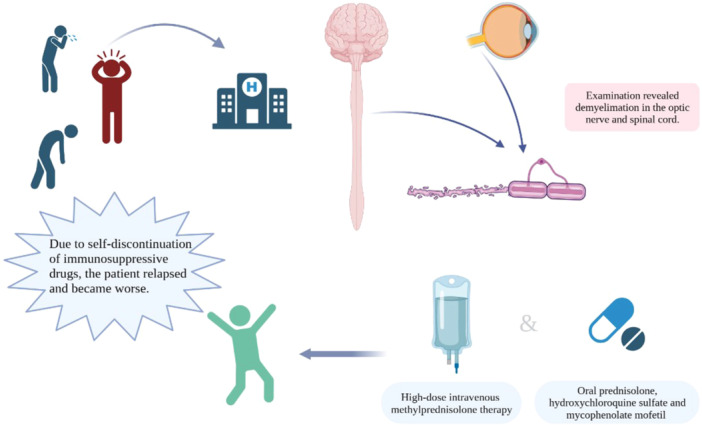

## 1 INTRODUCTION

Neuromyelitis optica spectrum disorder (NMOSD) is an immune‐mediated demyelinating disorder of the central nervous system that mainly affects the optic nerve and the spinal cord. NMOSD typically exhibits two age‐related manifestation peaks, affecting both younger and older patient groups. The core clinical syndromes of NMOSD include optic neuritis leading to loss of vision, myelitis‐caused spinal cord syndrome, and intractable nausea and vomiting.^1^ Sjogren's syndrome (SS) is classically defined as an autoimmune inflammatory disease that is clinically characterized by exocrine gland dysfunction, primarily resulting in dry eyes and dry mouth.^2^ However, SS often extends beyond the exocrine glands and severely affects other organs and systems, such as the lungs, kidneys, and nervous system.^2^ Several studies have demonstrated the association of NMOSD with autoimmune diseases such as SS and systemic lupus erythematosus (SLE).^1^, ^3^ The treatment of NMOSD comorbid with SS is based on the experiences from treating autoimmune diseases and/or NMOSD.^4^ However, reports on treatment outcomes in these cases are limited.

Here, we reported a girl with a diagnosis of “NMOSD comorbid with SS” who exhibited notable improvement after treatment with corticosteroids and immunosuppressive medication (prednisolone, hydroxychloroquine, and mycophenolate mofetil). However, a relapse occurred due to her voluntary discontinuation of the immunosuppressive drugs, leading to the development of a more severe form of the disease.

## 2 CASE REPORT

In October 2014, a 14‐year‐old Chinese girl was admitted to the Neurology Department of the Affiliated Hospital of Zunyi Medical University with complaints of fever, dizziness, dry mouth, weakness of lower limbs, and mental abnormalities for 1 week. During the patient's attack, her brain magnetic resonance imaging (MRI) revealed multiple lesions in the bilateral basal ganglia, corpus callosum, and left cerebellar hemisphere. Additionally, thoracic vertebra MRI indicated degeneration of the thoracic spinal cord, suggesting the presence of inflammatory lesions. Serological tests showed the patient was tested positive for anti‐SSA/SSB antibodies, anti‐RO‐52 antibodies, and anti‐nuclear antibodyanti‐nuclear antibody (ANA). Due to financial constraints, the patient did not undergo testing for aquaporin‐4 antibody IgG (AQP4‐IgG). However, considering the patient's clinical presentation and test results, the diagnosis of NMOSD comorbid with SS was established. The condition of the patient showed marked improvement after one cycle of high‐dose intravenous (IV) methylprednisolone therapy (1000 mg once a day for five consecutive days), followed by oral prednisolone (50 mg once a day) and hydroxychloroquine sulfate (200 mg twice a day). Regular dose adjustment and cyclophosphamide (CTX) injection were administered to control the condition.

In July 2015 (approximately 9 months after the initial diagnosis and treatment), the patient was re‐performed relevant tests for further regular treatment. Anti‐SSA/SSB antibodies, anti‐RO‐52 antibodies, and anti‐ANA were still positive. Ten months after treatment, MRI of the brain and thoracic vertebra indicated a significant reduction in lesions compared to the previous one, indicating the therapeutic efficacy of current treatments. However, as there were still intracranial lesions, immunosuppressive treatment with mycophenolate mofetil (MMF) (0.5 g bid) was added.

In December 2018 (approximately 4 years and 2 months after the initial diagnosis and treatment), the patient was admitted to the hospital again due to a sudden loss of vision in her right eye without pain. After careful inquiry, the patient reported that she had ceased taking the immunosuppressant for 1 year (from December 2017 to December 2018) due to side effects, such as irregular menstruation, nausea, and vomiting. [Correction added on 5 June 2025, after first online publication: The time period mentioned in the preceding sentence was revised from “January 2019 to January 2020” to “December 2017 to December 2018”.] One year after discontinuing treatment, pattern visual evoked potential (PVEP) showed that the latency of the right eye was approximately 35% longer than that of the left eye, and there was a significant reduction in the amplitude of the visual evoked potential peak (P100). MRI revealed multiple patches and spot‐like long T2 weighted imaging (T2WI) signal shadows in the white matter areas of the brain, and fluid‐attenuated inversion recovery (FLAIR) sequence showed a high signal. The right optic nerve exhibited a slight increase in thickness and higher signal. Subsequently, the patient's vision improved by using the same regimen. The patient's drug dosage remained unchanged compared to the previous regimen. Symptoms were alleviated when the same dosage and course of treatment were administered, gradually allowing for a return to normal. Following a complete course of treatment, the patient's symptoms were relieved. To reduce the side effects of drugs, we provided the patient with stomach protection, calcium supplementation, and maintenance of water and electrolyte balance.

**Figure 1:**
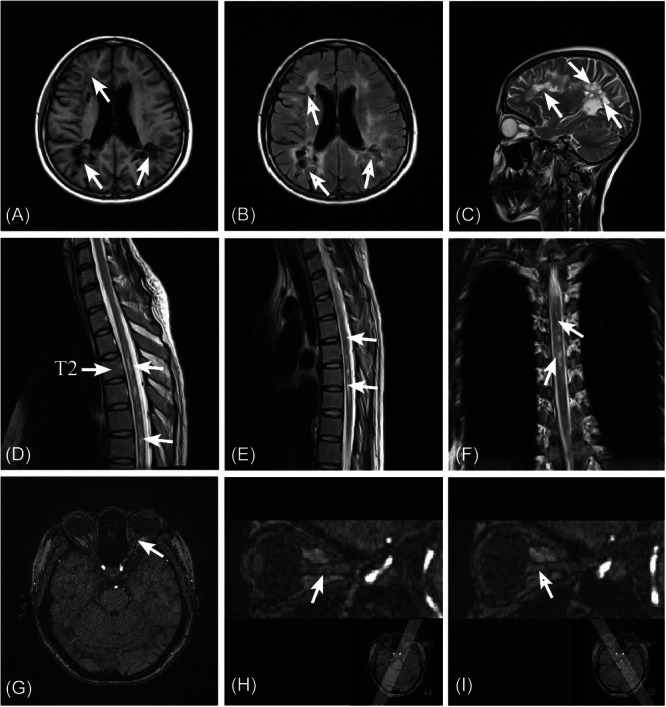
MRI images of brain and thoracic medulla (August 10, 2021, approximately 6 years and 9 months after the initial diagnosis and treatment). (A, C) There were multiple demyelinating plaques with heterogeneous signal in both cerebral hemispheres (white arrow). (B) Some areas in the FLAIR sequence were low‐intensity (white arrow). (D–F) In the upper thoracic segment (approximately flat chest level 2‐6), the spinal cord had a multi‐slice‐length T2 signal, and the contrast‐enhanced scan showed significant enhancement (white arrow). (G, H) The left optic nerve was slightly thickened (white arrow). (I) The right optic nerve showed no abnormality in its course, shape, or signal (white arrow).

However, the patient stopped taking the drug again for 1 year for the same reason. [Correction added on 5 June 2025, after first online publication: The preceding sentence was revised for clarity from “1 year later” to “for 1 year”.] In August 2021 (approximately 6 years and 9 months after the initial diagnosis and treatment), her symptoms were worse than ever, manifested by symmetrical weakness of both lower extremities, decreased position and vibration sensation, reduced tendon reflexes, positive Chaddock's sign, and diminished sensation below the level of chest (level 5). In addition, she had dryness in her right eye and impaired vision in the left eye. One year (from July 2020 to July 2021) after withdrawal from treatment, there was a significant increase in the brain lesions (Figure 1A–C) and thoracic medulla lesions (Figure 1D–F) compared to the previous examination, and slight thickening of the left optic nerve on MRI images of brain (Figure 1G–I).

**Figure 2:**
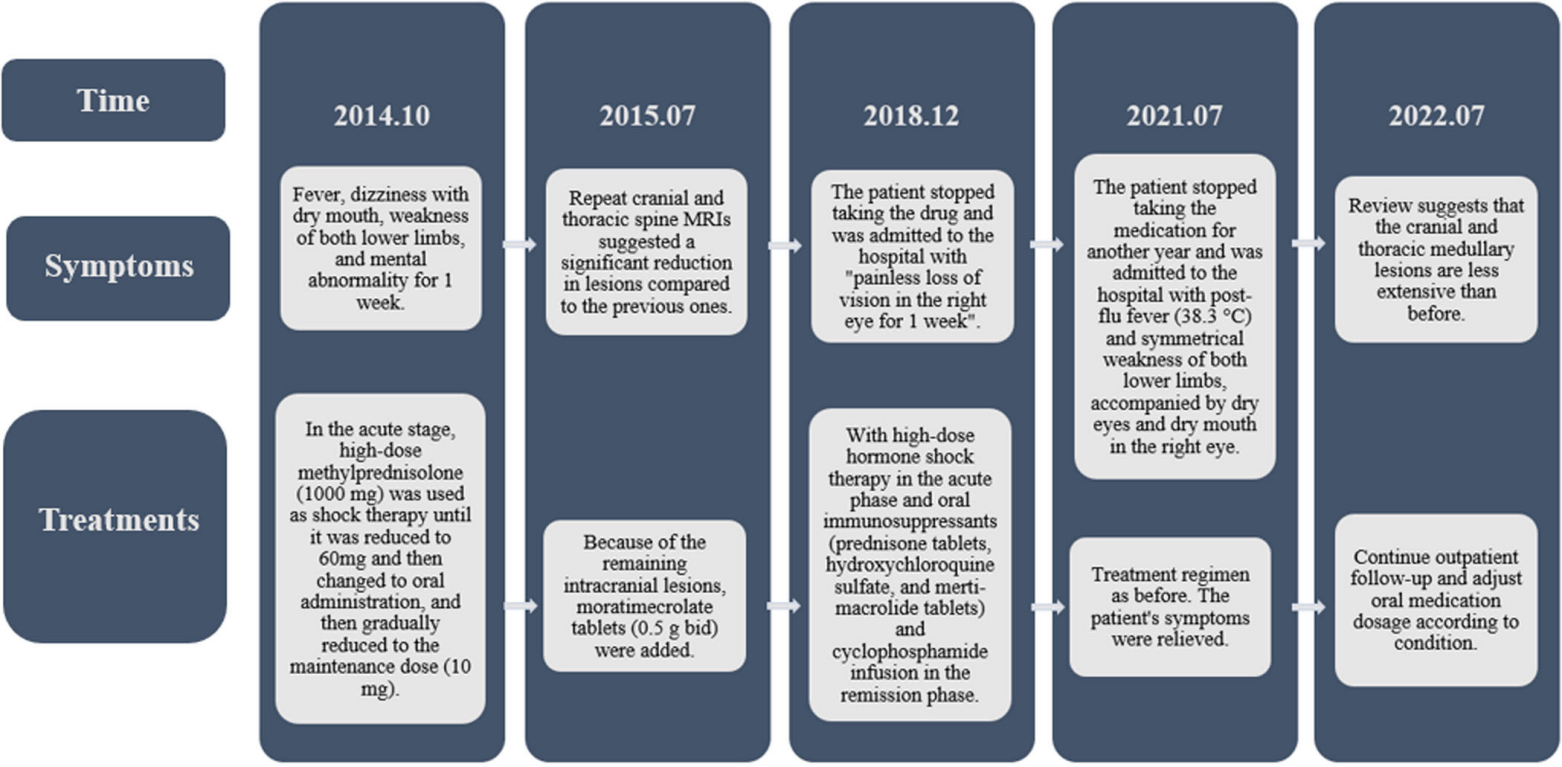
Flow chart of the course and treatment plan.

After admission, she was treated with immunosuppressive therapy. Two weeks later, her symptoms of lower limbs weakness, vision loss, dry mouth, and eyes had improved significantly. After discharge from the hospital, the patient received regular telephonic follow‐up. On July 21, 2022, the patient was still taking immunosuppressants regularly and being reviewed regularly, with no recurrence of symptoms. The course of the disease, the diagnosis and treatment program flow diagram are represented in Figure 2.

## 3 DISCUSSION

NMOSD is a rare group of recurrent autoimmune diseases of the central nervous system, primarily characterized by recurrent episodes of myelitis and optic neuritis.^5^, ^6^ In recent years, NMOSD has often been found to co‐exist with other autoimmune diseases, such as SS, myasthenia gravis, and SLE.^1^, ^3^ SS is a systemic autoimmune inflammatory disease that frequently results in damage to the peripheral nervous system and/or central nervous system.^4^, ^7^ The central nervous system involvement is mainly manifested by recurrent optic neuritis, focal neurological deficit, dyskinesia, or spinal cord lesions.^4^ Dean et al. noted that patients with rheumatic disease, who presented longitudinal pervasive transverse myelitis, recurrent optic neuritis, myelitis, or characteristic brainstem syndrome (such as intractable vomiting or burping) with characteristic MRI lesions, are likely to have NMOSD comorbid with their rheumatic disease.^8^

The recommended therapies for NMOSD and SS are often similar, with immunosuppression being the preferred approach. The first‐line immunosuppressants for NMOSD and SS include rituximab (RTX), MMF, and azathioprine (AZA), while the second‐line drugs include CTX, mitoxantrone, and natalizumab. Several recent studies have compared the efficacy of first‐line agents and found that RTX, AZA, and MMF can all effectively reduce recurrence and improve or stabilize disability, regardless of APQ4‐IgG status.^9^, ^10^ In addition, the European Federation of Neurological Societies recommends the use of CTX as second‐line treatment for acute NMOSD relapse, especially when NMOSD is comorbid with connective tissue diseases like SLE or SS.^6^ Nevertheless, the optimal drug regimen and treatment duration have not been determined. Depending on the severity of the disease, various therapies may be employed. High‐dose corticosteroid therapy is recommended as the first‐line treatment in the acute phase of NMOSD, usually involving IV administration of methylprednisolone at a dosage of 1 g per day for 3–5 days, followed by a gradual tapering to an oral maintenance dose. In cases where corticosteroids are less effective or if the disease recurs, plasmapheresis or IV immunoglobulin may be considered as alternative options. A retrospective study has suggested that although plasmapheresis is considered as a second‐line treatment, early systematic use of plasmapheresis in combination with corticosteroids, rather than corticosteroids alone, can minimize disability and hasten recovery in patients with the first episode of NMOSD.^11^ Compared with previous studies, this article highlights the importance of immunosuppressive therapy in treating NMOSD with SS.

In this particular case, disease stabilization and remission were successfully achieved after a relapse with the combination of steroid hormones, MMF, and CTX. In addition to the timely and effective treatment in the acute episodes, it's essential to continue treatment during remission to reduce the risk of relapse and delay the accumulation of disability. Immunosuppression often requires long‐term treatment to achieve these goals. Abrupt interruption of treatment in patients with NMOSD and SS may lead to more severe relapse, as illustrated in this case report. The degree of disability in patients with comorbid NMOSD and SS is associated with the accumulation of recurrent episodes, rather than disease progression. During a relapse of NMOSD, a subacute increase in symptoms reaches a plateau and then gradually subsides, usually without complete remission.^12^ During the course of relapsing, exacerbations and incomplete remissions cause an accumulation of disability, which has a profound impact on the quality of life of the patient.^5^ In contrast to other central system demyelinating disorders, NMOSD relapses tend to be more severe and almost irreversible, usually resulting in loss of vision, paralysis, bladder dysfunction, disturbance of consciousness, and even respiratory failure and death.^13^ A survey conducted in Germany showed that the health care expenditure in NMOSD patients was positively correlated with disease severity, while the health‐related quality of life was negatively correlated with disease severity.^14^ This demonstrates the importance of accurately assessing the likelihood and severity of relapse at an early stage of the disease, as timely diagnosis and treatment will bring about a significant improvement in the quality of life and reduce the financial burden for patients. Most current treatments focus on relapse prevention, as prevention of attacks is crucial in reducing permanent disability. Therefore, a long‐term treatment program should be initiated as soon as NMOSD is diagnosed. Another key is that there is no consensus on how long immunotherapy should last, with more recommendations even recommending lifelong treatment. Hence, in this case, the patient's abrupt discontinuation of the drug after a long period of immunotherapy was a wrong approach. Long‐term and continuous immunotherapy is a crucial factor in preventing recurrence and exacerbation of the disease.

Although immunosuppressive therapy acts fast, it brings about inevitable side effects such as peptic ulceration, liver and kidney impairment, opportunistic infections, osteoporosis and so on. Severe side effects often contribute to poor treatment compliance, and thus patients would abandon or stop treatment, consequently increasing the risk of relapse. Therefore, during formulation of treatment plans, careful consideration should be given to the potential side effects of drugs. In addition, patients should receive counseling about the possibility of side effects and the corresponding remedial measures. They should also be educated about the risks associated with treatment discontinuation, as this will lead to more serious relapses. Moreover, patients should promptly seek medical attention if they feel unwell to avoid interruption of treatment and adverse events.

## 4 CONCLUSION

Collectively, this report revealed that long‐term, continuous, and regular immunosuppressive treatment is essential for NMOSD comorbid with SS to reduce the risk of relapse and promote functional recovery. Clinicians should also be vigilant about the possibility of optic neuritis and myelitis in patients with NMOSD and SS to ensure early diagnosis, standardized treatment, and better prognosis.

## AUTHOR CONTRIBUTIONS

Hao Huang and Zu‐Cai Xu conceived and designed the study; Hao Huang made significant contributions to conception, acquisition of clinical information, analysis and interpretation; Man‐Min Zhu drafted the manuscript and assisted in the preparation of the figures; Chang‐Yin Yu reviewed the manuscript and offered suggestions. All authors read and approved the final version of the manuscript.

## CONFLICT OF INTEREST STATEMENT

Zu‐Cai Xu is Associate Editor of *Ibrain* Journal editorial board. He is not involved in the peer review and editorial decision‐making processes of this article. The remaining authors declared they have no competing interests.

## TRANSPARENCY STATEMENT

The authors affirm that this manuscript is an honest, accurate, and transparent account of the study being reported; that no important aspects of the study have been omitted; and that any discrepancies from the study as planned (and, if relevant, registered) have been explained.

## ETHICS STATEMENT

This case report was approved by the ethics committee of the Affiliated Hospital of Zunyi Medical University (Approval No: KLL‐2023‐239). The patient gave written informed consent for publication of medical information and images.

## Data Availability

The data that support the findings of this study are openly available upon reasonable request.
